# Research Trends in Isothermal Near-Net-Shape Forming Process of High-Performance Titanium Alloys

**DOI:** 10.3390/ma18030578

**Published:** 2025-01-27

**Authors:** Shuangjie Chu, Weiwei Huang, Gaofei Liang, Qingtong Meng, Xiangyu Zhou, Bo Mao

**Affiliations:** 1 School of Materials Science and Engineering, Shanghai Jiao Tong University, Shanghai 200240, China; chushuangjie@sjtu.edu.cn; 2 Baoshan Iron & Steel Co., Ltd., Shanghai 201900, China; 3 Baowu Special Metallurgy Co., Ltd., Shanghai 200940, China; lianggf@baosteel.com (G.L.); mengqingtong@baosteel.com (Q.M.); 741105@baosteel.com (X.Z.)

**Keywords:** near-net-shape forming, isothermal forging, titanium alloys, microstructure evolution

## Abstract

Titanium alloys find extensive applications in aviation, maritime, and chemical engineering applications. Nonetheless, these alloys encounter significant challenges during the conventional forging process, which include high deformation resistance, limited processing temperature ranges, and inhomogeneous microstructure. Isothermal forging, as a near-net-shape forming technique, can alleviate the microstructural inhomogeneity caused by deformation dead zones in conventional forging, thus enabling the direct production of complex shapes. This process enhances the overall performance and utilization of materials while reducing manufacturing costs. This paper comprehensively reviews how isothermal near-net-shape forging process parameters influence the intricate microstructure and essential properties of titanium alloys. The unique properties of isothermal forging applied to high-performance titanium alloys are also discussed in depth, and the intricate interplay between process parameters and the microstructure and properties of recoloration is clarified. That is to say, temperature is a vital element influencing the phases and microstructure of titanium alloys during the forming process. Grain size, microstructural homogeneity, and phase transformation are influenced by the strain rate, thereby affecting the plasticity, fracture toughness, and strength of titanium alloys. The extent of deformation significantly governs the grain size, the thickness of secondary α phase, dynamic recrystallization, and primary α phase. Cooling rate affects the grain size and precipitates, contributing to grain refinement. The frequency of isothermal forging affects the grain refinement and microstructural uniformity of titanium alloys. Finally, this paper summarizes the scientific questions that remain unresolved in this field and outlines future research directions to promote the further development of isothermal near-net-shape forging processes and facilitate the broader industrial applications of high-performance titanium alloys and other difficult-to-form alloys.

## 1. Introduction

**Figure 1:**
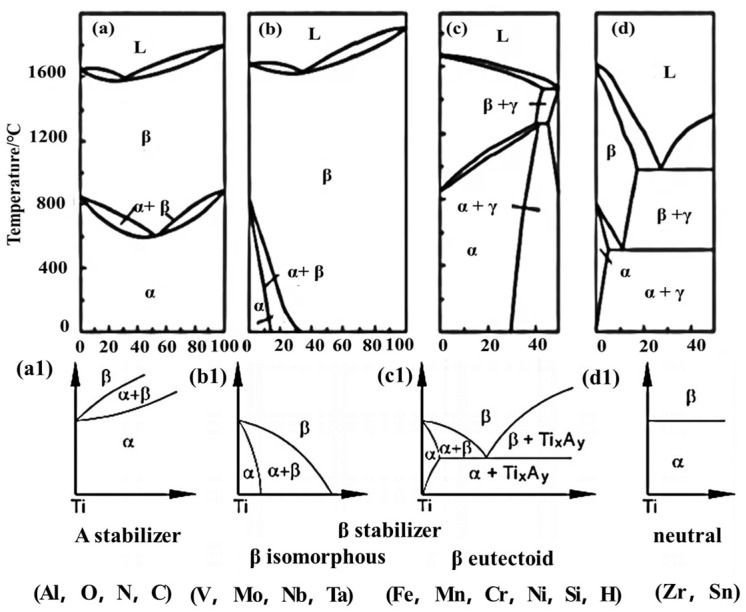
The four typical phase diagrams of titanium alloys: (**a**,**a1**): Phase diagram of α stable elements; (**b**,**b1**): Phase diagram of β isomorphic stable elements; (**c**,**c1**): Phase diagram of β eutectic stable elements; (**d**,**d1**): Phase diagram of a neutral stable element; [4,12].

Titanium is often hailed as the “Metal of the 21st Century”, “Metal of the Space”, and “steel of the future”. Titanium-based alloys have been employed in the production of complex load-bearing components for navigation [1] aviation [2], chemical [3], and other related equipment to reduce weight, thereby achieving product quality while reducing the amount of material used. The characteristics of titanium alloys are determined by the relative proportions of α and β phases, as well as their microstructural configurations. Table 1 summarizes the processing techniques and corresponding performance advantages of each microstructure [4,5,6,7,8]. It can be observed that different microstructures require distinct forging processes, which subsequently result in varying mechanical properties. Typical structures include equiaxed microstructure, duplex microstructure, basketweave, and lamellar structures. The microstructure is influenced by composition, deformation processes, and heat treatment [9]. The distinction lies in the content and morphology of the α phase. When the α phase content exceeds 40%, the microstructure is equiaxed, whereas a content of approximately 30% corresponds to a bimodal microstructure. Both the basketweave and lamellar microstructures exhibit an α phase content of zero. The basket-weave structure is characterized by α lamellae with a low aspect ratio and an interwoven arrangement, resembling a basketweave. In contrast, the lamellar structure retains intact original β grains, within which the α phase appears primarily as straight, orderly lamellae, often forming bundles. The microstructure of each type varies depending on the alloy composition [10]. Leveraging the polymorphic transformation characteristics of titanium, various phase-stabilizing elements can be added to alter the phase transformation temperature and phase composition [11]. The typical phase diagram is illustrated in Figure 1. Based on the effects of alloying elements on the β-transus temperature of titanium, four types of phase diagrams can be classified as follows: α-stabilizing elements, β-stabilizing elements, and neutral elements [12]. According to the phase composition after annealing, titanium alloys can be categorized into α-type, β-type, and α + β-type alloys. The performance of titanium alloys depends on the relative proportion of α and β phases as well as their microstructural morphology. The β phase exhibits high strength, numerous slip systems, and ease of plastic deformation, making it the primary matrix for high-strength titanium alloys [13]. The α phase features tightly packed atomic arrangements and limited slip systems, leading to excellent heat resistance and creep resistance. Therefore, high-temperature titanium alloys are typically α or near-α alloys [14]. Cooling methods can lead to different precipitation phases in titanium alloys after forging, including α′, α″, and ω phases, which can be removed through heat treatment. Thus, with a fixed composition, the microstructural configuration of titanium alloys is predominantly influenced by the processing methods employed.

The difficulty in the machining of titanium alloys is also one of the factors limiting their applications. Titanium alloys subjected to large loads or complex stress conditions need to be forged; however, these alloys encounter problems such as high deformation resistance, a limited forging temperature range, and poor microstructural homogeneity in conventional forging processes [15]. Even minor temperature fluctuations can lead to dramatic changes in deformation resistance. For TC4 (Ti-6Al-4V) alloy, each 100 °C reduction in forging temperature leads to an approximate 200 MPa increase in deformation resistance, which places very stringent restrictions on the processing conditions. Therefore, if titanium alloys are processed using conventional die forging, the required tonnage of the equipment is often quite large [16]. Additionally, the strict control of forging temperature is one of the reasons that makes titanium alloys challenging to forge [17]. Titanium alloys are hot processed at 30–100 °C, that is, below the β transition temperature, which exhibits good plasticity and workability, accommodating the deformation requirements of the forging process. However, if the heating temperature is inadequate, deformation resistance significantly rises, leading to poorer hot workability and defects such as surface cracking [18]. Heating above the β transition temperature promotes grain growth, adversely impacting the alloy’s plasticity and causing parts to experience early failure during experiments. This complicates the subsequent heat treatment processes, making it difficult to achieve uniform deformation and control of microstructure [19]. Deformation at elevated temperatures is likely to result in overheating wherein material deformation occurs through the relative sliding of grains at their boundaries. High temperatures cause the grain boundaries to behave like a fluid, exhibiting viscous properties, which leads to the initiation and propagation of microcracks, increasing processing difficulty and costs [20]. Thus, tightly controlling the forging temperature range is the foremost step in ensuring the quality of titanium alloy forging. Titanium alloys exhibit low thermal conductivity, and significant deformation at high strain rates requires consideration of the thermal effects of deformation and increasing temperature. The effect of temperature rise can cause local temperatures to exceed the forging temperature range of the alloy, resulting in poor microstructural uniformity and worsening the mechanical properties of the component [21]. Hence, there is an urgent need for a near-net shape forging process that can reduce deformation resistance, accurately control the forging temperature, improve the uniformity of the microstructure, shorten the production cycle, and reduce production costs.

**Figure 2:**
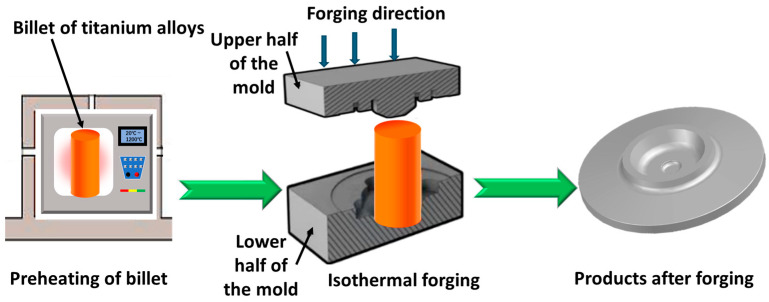
Schematic diagram of the isothermal forging of titanium alloy.

The introduction of the isothermal forging process enables the precise machining of complex shapes while maintaining material performance [22]. The fundamental principle of isothermal forging technology involves heating the mold to a temperature that matches or is close to the deformation temperature of the billet and achieving near-net-shape forging by controlling pressure during low-speed deformation [23]. As shown in Figure 2. The titanium alloy billet is transferred to the isothermal forging equipment when it is heated to the forging temperature, at which time the die has been heated to the same equipment as the billet. By controlling the forging rate, the titanium alloy is plasticized into the desired shape, completing the whole near-net-shape forging process. In the forming process, titanium alloys can deform plastically in a uniform manner while maintaining lower deformation resistance, thus addressing poor forming performance and the chilling effects caused by the mold [24]. Under quasi-static temperature conditions, dynamic recrystallization happens uniformly, preventing the issues of residual stress and poor microstructural uniformity arising from the temperature gradients and uneven internal deformation of the conventional forging process. This contributes to the improved structural stability and lifespan of titanium alloy forgings. Therefore, this process enables the production of near-net-shaped complex structures and significantly improves the dimensional accuracy and performance of forgings [25]. Additionally, based on Pascal’s law, only low-power equipment is needed to generate several times higher pressures, thereby facilitating the forging of large and complex structural components. Controlling the forging speed enables the production of high-precision components with intricate shapes, greatly minimizing energy consumption and material waste. During isothermal forging, the grains of titanium alloys gradually refine and become denser under constant temperature and lower strain rates [26]. Grain refinement helps resist dislocation and crack propagation, reduces porosity and defects within the material, and further enhances the comprehensive performance of titanium alloys. Thus, for titanium alloys that have high raw material costs and considerable deformation resistance, the use of isothermal forging to achieve near-net-shape forming can strengthen the internal structure to meet performance requirements while conserving raw materials and lowering processing costs [27].

Currently, isothermal forging technology has been utilized in the manufacturing of integral blade discs for aerospace engines, contributing to the overall weight reduction in aircraft. TiAl alloys possess particularly remarkable high-temperature properties, but their poor hot workability renders standard die forging ineffective [28]. This results in high costs for forging, welding, and machining. Nevertheless, the application of isothermal forging has successfully allowed TiAl alloys to be utilized as high-pressure compressor rotor blades in military engines [29]. The components where titanium alloys can best leverage their mechanical performance in the aircraft industry are the dual-rotor engines, the low-pressure compressor rear section, the high-pressure compressor front section, and the mid-pressure compressor of three-rotor engines, where temperatures are moderate and the rotors bear significant loads. These components tend to exploit high-strength Ti alloys such as Ti-6426 (Ti-6Al-2Sn-4Zr-6Mo) and Ti-17 (Ti-5Al-2Sn-2Zr-4Mo-4Cr) to enhance resistance to fracture [30]. Typically, β die forging is used to obtain a basketweave structure; however, controlling the final forging temperature during β forging is difficult, which may drop into the α + β region, resulting in an uneven microstructure with equiaxed α phase along grain boundaries and lamellar structures within the grains. By applying isothermal forging to β forging, a mesh basket structure with uniform tissue distribution can be obtained, and the forging of high-performance titanium alloys can be realized [31]. These greatly demonstrate the superiority of the isothermal forging process in the precision manufacturing and forming of high-performance titanium alloys.

During the isothermal forging process, the microstructural characteristics of titanium alloys are closely linked to their mechanical properties, with factors such as phase composition, morphology, distribution, and grain size significantly influencing the material’s toughness and strength [32]. The design and optimization of processing and heat treatment procedures can significantly influence the microstructure, subsequently affecting the mechanical properties [33]. The statistics show that the majority of the final-stage blades in aircraft fail due to fatigue, jeopardizing the overall safety and reliability of the system. Harsh operating environments impose greater requirements on the fatigue resistance of turbine blades [34]. Isothermal forging induces plastic deformation at ultra-low strain rate levels, and the parameters of the forging process significantly impact the fatigue resistance and other properties of the alloys, directly threatening their safety and reliability. Therefore, through the meticulous selection of the forming process parameters, titanium alloys can achieve diverse microstructural characteristics.

In-depth research on the isothermal forging process parameters is essential for understanding the performance control and microstructural evolution of titanium alloys. Therefore, in this paper, the effects of important factors such as forging temperature, deformation, strain rate, and cooling rate of titanium alloy on the microstructure and properties of titanium alloy during isothermal forging are reviewed. A comprehensive understanding of the inherent microstructural characteristics of titanium alloys and isothermally forged titanium alloys is crucial for accelerating the development of innovative and high-performance titanium alloy components. Furthermore, refining processing methods to regulate the microstructure of these materials is vital for unlocking their potential capabilities.

## 2. Analysis of the Influence of Isothermal Near-Net Shape Forging Process Parameters

### 2.1. Temperature of Isothermal Forging

**Figure 3:**
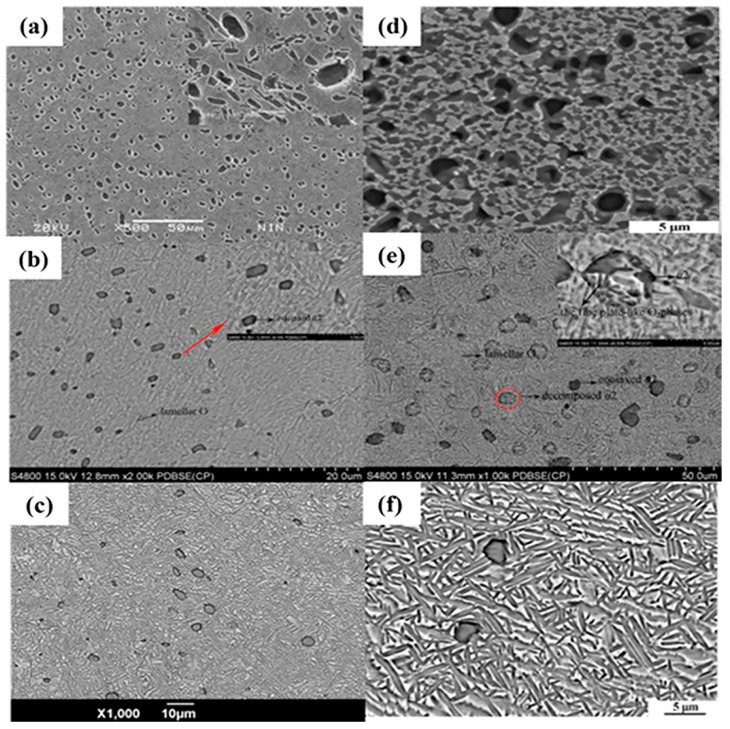
SEM mages images of Ti-22Al-25Nb alloy: (**a**) 980 °C, (**b**) 1040 °C, and (**c**) 1060 °C.; Solution-treated, aged tissues: (**d**) 980 °C, (**e**) 1040 °C, and (**f**) 1060 °C [38].

Temperature affects the phase transitions in titanium alloys, creating various phases and microstructural forms, thereby impacting the mechanical properties and areas of application. Typically, in the single-phase region, the forging temperature is at least 10 °C above the phase transition point, and sometimes up to 50 °C above it. In the two-phase region, the forging temperature is controlled to be 30~50 °C below the phase transition point [4]. Zhou et al. [35] proposed an effective method for preparing titanium alloys with a three-phase microstructure, achieved through near-β forging and high/low low-temperature strengthening heat treatment, resulting in 20% equiaxed α phase, 50–60% basket-weave layered phase, and the remaining β β-transformed structure. Shi et al. [36] explored the microstructural variations of TC21 alloy (Ti-6Al-2Sn-2Zr-2.5Mo-2Nb-1.5Cr-xSi) by controlling the isothermal forging process at 900 °C, 940 °C, 950 °C, 970 °C, and 980 °C. The findings indicated that at 940 °C and below, only a limited amount of primary equiaxed α phase was observed along withside coarse α plates, with the quantity of primary equiaxed α phase decreasing as the forging temperature increased. Yang et al. [37] noted multiple deformation modes in samples of Ti-23.1Nb-2.0Zr-1.0 alloy, including dislocation slip, twinning, stress-induced ω phase transformation, and stress-induced martensitic phase transformation. The forging equipment is a 630 tonneton hydraulic press, which is capable of forging at a constant strain rate. The forging parameters set in the experiments included die temperatures of 900 °C and 940 °C, forging temperatures of 900 °C, 940 °C, 950 °C, 970 °C, and 990 °C, strain rates of 5.5 × 10^−4^ s^−1^ and 1 × 10^−2^ s^−1^; and the height reduction was of 60% for each forging. Notably, in samples subjected to higher strain rates and lower temperatures, the first observed transformation was the stress-induced ω phase transition. TA15 titanium alloy (Ti-Al-Mo-V-Zr-Fe) was first heated to 960 °C and held at a rate of 12 °C/min for 20 min to form a microstructure containing a certain amount of incipient α phase. After the samples were subsequently cooled to 870 °C and 810 °C, the samples were deformed at a strain rate of 0.1 s^−1^ until reaching a total strain of 0.7. The dynamic transition of the α_p_ phase to the β phase during deformation at 870 °C significantly reduces the volume fraction of the primary α phase as the driving force for the stress difference at high temperatures outweighs the energy barrier for the phase transition. In contrast, at 810 °C, the strain-induced β to α_p_ phase transition and the dynamic α_p_ to β phase transition canceled each other out, resulting in little change in the volume fraction of the main α -phase. The findings indicated that high high-temperature deformation significantly reduced the volume fraction of the main α phase. Wang et al. [38] studied the Ti-22Al-25Nb alloy by conducting isothermal forging at the following three temperatures: 980 °C, 1040 °C, and 1060 °C. After forging, the samples exhibited equiaxed structure, duplex structure, and dual-phase layered orthogonal structure (Figure 3a–c). Different microstructures were achieved following the different heat treatment temperatures (Figure 3d–f). Ultimately, mechanical performance tests indicated that the alloy forged at 1060 °C had the highest strength but the poorest ductility, whereas the alloy forged at 980 °C showed the best ductility but the lowest strength. Thus, temperature is found to be a crucial factor affecting the microstructure of titanium alloys.

**Figure 4:**
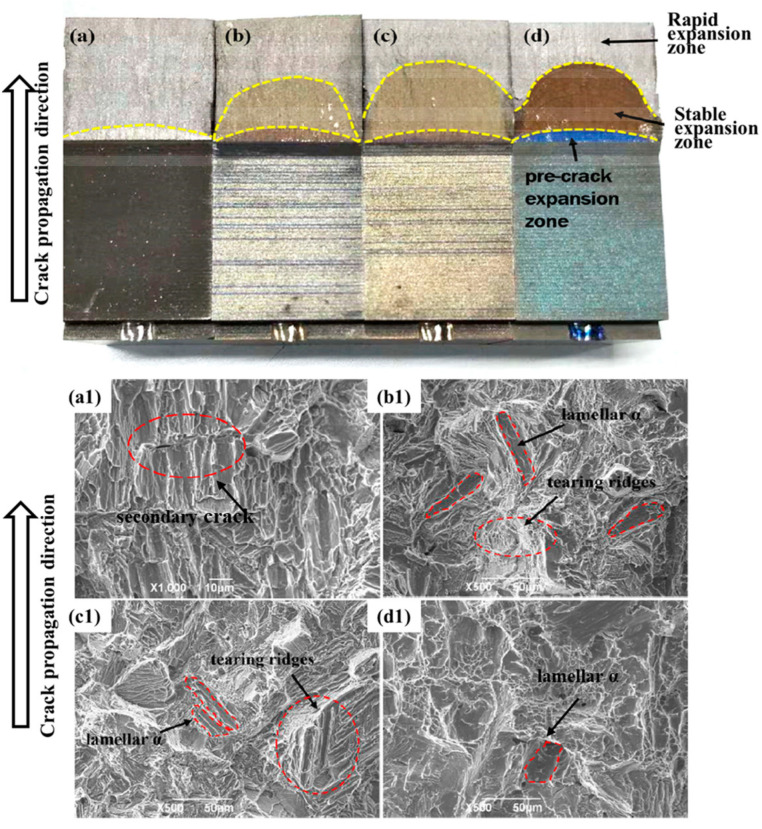
Macroscopic fracture samples of Ti-Al-Sn alloy: (**a**) Raw raw material,; (**b**) 200 °C; (**c**) 400 °C; (**d**) 600 °C.; Microscopic fracture images: (**a1**) Raw raw material; (**b1**) 200 °C; (**c1**) 400 °C; and (**d1**) 600 °C [39].

Temperature impacts the grain size and morphology of titanium alloys during forging, which subsequently affects their performance. Isothermal forging results in smaller grains and primary α phases compared to conventional die forging, which aids in enhancing the material’s plasticity and toughness. Under high-temperature conditions, the refined grains and uniform structure can better withstand thermal stress and creep deformation, thereby improving the material’s high-temperature strength and service life. Jia et al. [39] analyzed the fracture mechanisms and tensile properties of Ti-Al-Sn (Ti60) alloys at 200 °C, 400 °C, and 600 °C, respectively. The findings indicate that high temperatures alleviate dislocation accumulation and activate more slip systems, thereby enhancing deformability and ductility. Fracture toughness increases with increasing temperature, showing the following three stages: the pre-crack propagation zone, stable crack propagation zone, and rapid crack propagation zone, which are marked with yellow curves in the cracks, as shown in Figure 3. In unheated titanium alloys, the main crack alters its direction during propagation, and secondary cracks tend to form at the α boundaries, marked with red circles in Figure 4a1. As the temperature increases, the number of accessible slip systems also rises, leading to isolated lamellar α phases on the fracture surface, as illustrated in Figure 4b1. Due to the presence of grain orientations that are relatively difficult to deform, more tear ridges become evident on the fracture surface, especially within the lamellar α phase in Figure 4c1. Ultimately, the lamellar α morphology on the fracture surface disappears in Figure 4d1. This is because, at higher temperatures, more slip systems can be activated, significantly enhancing ductility. Conversely, the secondary α phases and residual β phase boundaries in the β β-transformed structure pose significant obstacles to slip, complicating the deformation process and leading to a substantial increase in strength. Zhang et al. [40] found that the strength and ductility of the innovative metastable β titanium alloy Ti-6Mo-5V-3Al-2Fe are greatly influenced by the average width and spacing of the secondary α phases. Therefore, to enhance the alloy’s strength and toughness while minimizing the secondary α phase and avoiding grain coarsening, higher forging temperatures should be selected whenever possible.

During the forging of titanium alloys, identifying a suitable temperature range is crucial to ensure that the forged components exhibit excellent mechanical properties. The effects of isothermal forging temperature on the structure and properties of titanium alloys are multifaceted, encompassing grain refinement, improved uniformity, and enhancements in mechanical properties such as strength, plasticity, and toughness. In practical applications, it is essential to select appropriate forging temperatures and process parameters based on the specific materials and application contexts to achieve optimal material performance. Furthermore, different titanium alloy compositions have distinct requirements for forging temperatures, necessitating new research aligned with emerging materials to determine the most suitable temperature range.

### 2.2. Strain Rate of Isothermal Forging

During hot deformation, strain rate affects grain size, microstructural uniformity, and phase transformation processes, impacting the plasticity, fracture toughness, and strength of titanium alloys [41]. The strain rate for the isothermal forging of titanium alloys typically ranges from 0.01 to 0.0001 s^−1^. Yang et al. [42] performed isothermal forging with Ti-6.5Al-2Sn-4Zr-4Mo-1W-0.2Si (BT25y) alloy bars of 270 mm diameter (working load of 6300 kN) in a four-column hydraulic press, with the die temperature set at 930 °C and the billet temperature at 960 °C. At the same time, the strain rate of the titanium alloy was controlled at a deformation of 60% of the deformation for the experiments of 1 × 10^−1^ s ^−1^, 1 × 10^−2^ s^−1^, and 1 × 10^−3^ s^−1^ for the experiments. The findings indicate that lowering the strain rate results in coarser lamellar α phase and indistinct β phase grain boundaries, enhancing the alloy’s microstructural uniformity. Under high strain rates, the fracture toughness of densely intertwined α clusters and thick α layers at grain boundaries achieves a peak value. Additionally, as the strain rate is decreased, the alloy’s elongation initially rises and then falls. Moreover, the alloy’s fracture mode also changes from quasi-cleavage fracture to ductile fracture and then to mixed brittle fracture mode as the strain rate progressively rises. Yu et al. [43] investigated the microstructure and comprehensive performance of the Ti-6Al-4V alloy during isothermal forging. The findings demonstrate that finer microstructures and high tensile strength can be obtained at moderate deformation and high strain rates. During high-temperature deformation, there is a notable interaction between the deformation temperature and the extent of deformation or strain rate. Particularly, when deforming at temperatures just below the β phase transformation point, the plasticity and tensile strength are sensitive to all process parameters, including the interactions. Li et al. [44] performed isothermal forging of Ti-45Al-10Nb alloy at 1250 °C using a low strain rate of about 10^−4^ s^−1^ to achieve 70% effective height reduction. This is combined with finite element simulations of the dynamic recrystallization behavior of titanium alloys at different regional strain rates during isothermal forging, and it is concluded that varying the strain rate can produce different recrystallization structures, thus providing a new method for fabricating materials with a gradient distribution.

**Figure 5:**
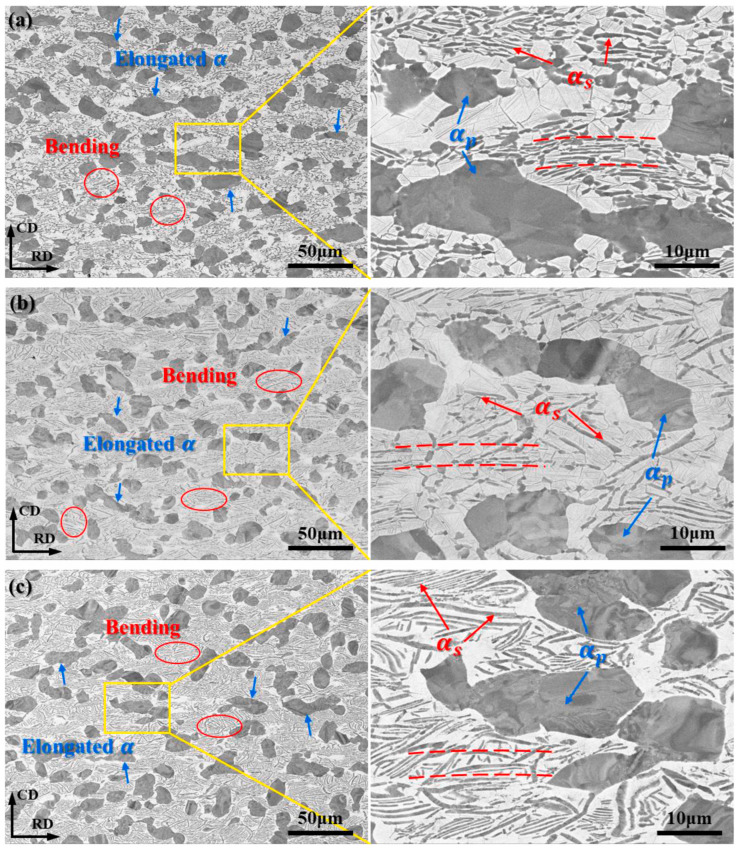
Microstructure of the Ti-6.5Al-3.5Mo-1.5Zr-0.3Si alloy at diverse strain rates: (**a**) 0.01 s^−1^, (**b**) 0.1 s^−1^, and (**c**) 1 s^−1^ [46].

Variations in strain rates can induce phase transformations, leading to varied microstructures [45]. Chai et al. [46] explored how different deformation strain rates affect the microstructure evolution of the α/β dual-phase Ti-6.5Al-3.5Mo-1.5Zr-0.3Si (TC11) alloy. In Figure 5, the primary equiaxed structure (α_p_) and the lamellar microstructure (α_s_) displayed substantial alterations compared to the original microstructure, as illustrated by the red ellipses and dashed lines. As the strain rate increases, the alloy’s elongation also rises; concurrently, the breakage of the lamellar structure becomes pronounced, causing the formation of numerous short rod-shaped α phases.

Notably, at a deformation strain rate of 0.01 s^−1^, the thickness of the lamellar α phase reaches its maximum, exceeding that of the initial lamellar thickness. This phenomenon occurs at a low strain rate, which extends the duration of deformation and provides ample opportunity for the lamellar structure to fracture and grow. At lower strains, the flow stress rises quickly, peaking during work hardening. As strain progresses, dynamic softening results in a gradual reduction in flow stress, which ultimately stabilizes over time.

Titanium alloys exhibit noticeable changes in grain size at different strain rates. Du et al. [47] examined the effect of strain rate on microstructure evolution during the subtransverse superplastic deformation of the BT25 (Ti-6.5Al-2Zr-2Sn-2Mo-1W-0.2Si) alloy, exhibiting that high strain rate deformation elongates both the α and β phases, while low strain rate deformation results in the coarsening of α grains and spheroidization of the β phase. Tong et al. [48] performed isothermal forging of the near-β type TB6 (Ti-10V-2Fe-3Al) titanium alloy within the β single-phase region. Their study demonstrated that the deformation rate is the key factor influencing grain size. At higher temperatures, grain size increases significantly with decreasing strain rate, but different texture types are present after solution heat treatment. Li et al. [49] developed a model for the dynamic recrystallization of the Ti-5Al-2Sn-2Zr-4Mo-4Cr alloy to study grain variations under different strain rates. It was found that the β grain size in titanium alloys was increased as the strain rate decreased.

**Figure 6:**
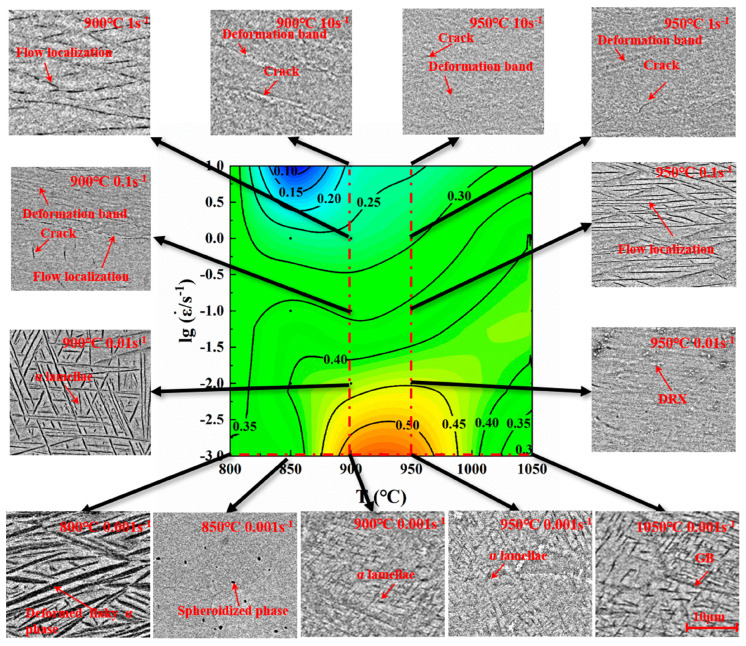
Characterization of the microstructure of a typical region in the thermal processing diagram of the Ti-6Al-2Sn-4Zr-6Mo alloy [49].

The mechanisms of deformation in titanium alloys differ under varying strain rates. With increasing strain rates, the major controlling factors for alloy deformation are in the following order: the diffusion-controlled creep process, thermally activated dynamic recovery and recrystallization processes [50,51,52], and the shear process influenced by localized heating. Adiabatic shear bands represent a common failure mode for materials subjected to high strain rates. Xue et al. [53] examined the hot processing map for the Ti-6Al-2Sn-4Zr-6Mo (TC19) alloy and found that strain rate has a primary influence on its thermal deformation mechanisms. Figure 6 illustrates the SEM images at different temperatures and strain rates. At a strain rate of 0.001 s^−1^, the lamellar α phase in the structure is gradually spheroidized with increasing temperature. As the strain rate increases, cracks and more unevenly distributed lamellar α phases also progressively appear in the microstructure. Furthermore, Zhang et al. [54] examined the behavior of deformation-induced α/β phase transformations in the Ti-6Al-2Zr-1Mo-1V alloy during hot processing, revealing that α_p_ can convert into the β phase under hot deformation conditions. Moreover, excessively high strain rates can result in incomplete recrystallization of the material at high deformation temperatures, leading to microstructural inhomogeneity and stress concentration during deformation [55]. Consequently, the processes governing microstructural evolution in titanium alloys are highly complex. Table 2 summarizes some of the microstructural evolution patterns of titanium alloys to provide partial references for studying the deformation process of isothermally forged titanium alloy.

At high strain rates, microstructural nonuniformity and stress concentration can occur along with changes to the grain structure, which in turn influence the mechanical performance. Consequently, higher strain rates make the fracture mode of titanium alloys more prone to brittle fracture. Uniform structure and proper grain size can reduce crack initiation and propagation, thereby improving the fracture toughness and fatigue life of the material. Therefore, a relatively low strain rate should be selected to ensure the uniformity of the structure and the refinement of the grains, such as controlling it within the range of 0.001 to 0.0001 s^−1^.

### 2.3. Deformation of Isothermal Forging

**Figure 7:**
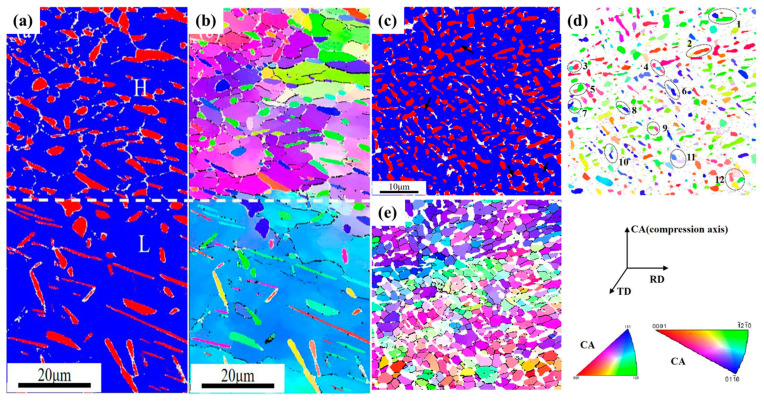
The EBSD results for the Ti-5Al-4Mo-4Cr-2Sn-2Zr alloy deformed to height reductions of 20%: (**a**) phase diagram and (**b**) inverse pole figure. The EBSD results for material deformed to height reductions of 60%: (**c**) phase diagram, (**d**) inverse pole figure of alpha phase, and (**e**) inverse pole figure of the β phase [57].

Deformation magnitude influences the amount of the primary α phase, the thickness of the secondary α phase, and the dynamic behavior of recrystallization. As the deformation amount during isothermal forging increases, the content of the primary α phase and the thickness of the secondary α phase in the titanium alloy initially show an increasing trend and then a decreasing trend. This occurs because of the increased deformation, which facilitates dynamic recovery and recrystallization, impacting the microscopic morphology and distribution of the phase. He et al. [56] explored the influence of 30%, 50%, and 80% deformation amounts on the microstructure and mechanical properties of the Ti-6.5Al-2.2Mo-2.2Zr-1.8Sn-0.7W-0.2Si (BT25) titanium alloy. During isothermal forging, as the deformation amount rises, the volume fraction of spheroidized α phase ranges from 16 to 40%, 40 to 76%, and 76 to 88%, respectively. The increase in the volume percentage of spheroidized α phase is responsible for the decrease in effective slip lengths, thereby enhancing tensile strength. Xu et al. [57] explored the impact of deformation amounts on the microstructure and crystallographic orientation of the Ti-5Al-4Mo-4Cr-2Sn-2Zr (TC17) titanium alloy during isothermal deformation. The research indicates that at low deformation levels, dynamic recovery serves as the main mechanism for the β phase. At larger deformations, the accumulation of distortion energy and dislocations promotes the separation of the lamellar α phase. EBSD images illustrate the results of material deformation at 20%, as shown in Figure 7a,b. α phase is marked as red and β matrix is blue. The H region and L region are marked as areas of large and small deformation, respectively. In the H region, the α plates are separated and achieve a nearly fully spheroidized structure. The α phase is still the same as the layered original structure found in the L region. Refined β grains form in the H region, while the initial β grains are preserved in the L region. At a deformation level of 60%, large deformation disrupts the Burgers orientation relationship between the grains, as illustrated in Figure 7c–e. Large deformation leads to globular α and β matrix, and β phase is formed due to deformation-induced low-angle and high-angle boundaries. Due to the cutting effect of the β phase, the α plate rotates asynchronously, eventually causing the splitting of the α plate boundaries, preparing for the formation of spheroidized α phase. Wang et al. [58] conducted an in-depth study on the factors affecting the final lamellar α phase of the Ti6242S titanium alloy. The study reveals that larger deformations produce greater distortion energy, facilitating the nucleation and growth of the lamellar α phase, thereby increasing its volume fraction. The increase in deformation will increase the thickness of the α phase, but the volume fraction of the α phase increases first and then decreases. At different deformation amounts, the crystal phase transformation and recrystallization behavior in titanium alloys are also different. For instance, a smaller deformation amount might result in microstructural evolution primarily governed by recovery, while a larger deformation amount may facilitate partial or complete recrystallization of the β phase, along with changes in the morphology of the primary α phase. The larger deformation in the isothermal forging process is beneficial to the occurrence of dynamic recrystallization, thus refining the grain structure and making the microstructure of titanium alloy more uniform and finer.

The extent of deformation significantly influences the yield strength and tensile properties of titanium alloys. Initially, an increase in deformation enhances tensile strength; however, beyond a certain point, tensile strength begins to decline, while plasticity exhibits an opposing trend. This behavior is attributed to the initial enhancement of the material’s density and grain boundary strength, which can be compromised by excessive deformation, potentially leading to structural damage and stress concentration. Zhang et al. [59] investigated the tensile properties of TiBw/Ti60 composites at 30%, 50%, and 70% deformation levels. The results indicated that, at room temperature, the tensile strength increased by 9.76%, 10.56%, and 3.91%, respectively, compared to the raw material. At 600 °C, the strength increased by 12.58%, 11.69%, and 6.22%, respectively. Notably, the sample deformed to 50% exhibited superior high-temperature mechanical properties with an ultimate tensile strength of 781.38 MPa and an elongation of 10.01% at 600 °C. Therefore, effective control of deformation is crucial for enhancing the mechanical properties of titanium alloys. Xu et al. [60] assessed the fracture toughness of isothermally forged titanium alloys by varying the deformation of Ti-17 alloy from 0% to 30% and 50%. Their findings revealed that the lamellar α phase structure contributes to crack bifurcation and increases the tortuosity of the crack propagation path, thereby elevating both the total length of the fracture and the material’s fracture toughness. Additionally, the volume fraction of the spheroidized α grain structure is positively correlated with the degree of deformation while the lamellar α phase decreases, leading to reduced fracture toughness. Increased deformation also enhances the material’s impact toughness, as it improves microstructural uniformity and reduces internal defects, thereby bolstering impact resistance.

The amount of deformation is a critical factor in controlling the dynamic recrystallization of alloys, which indirectly affects the tensile properties and yield strength of the material. Recent research indicates that during substantial deformation, small-angle grain boundaries increasingly transform into large-angle grain boundaries, facilitating continuous dynamic recrystallization. Additionally, many metastable titanium alloys exhibit discontinuous dynamic recrystallization during the forging process. Therefore, the design of the deformation amount should focus on maximizing single-pass deformation while avoiding the risk of cracking. Therefore, the strain is controlled within the range of 60~80%.

### 2.4. Cooling Rate of Isothermal Forging

Controlling the cooling methods and rates post-forging can significantly enhance the grain refinement effects of the forging process. A faster cooling rate results in a lower actual crystallization temperature of the metal, which in turn increases the nucleation position, resulting in a denser structure and smaller grain size. With the decrease in grain size, the number of grain boundaries increases. The grain boundaries significantly enhance the bonding strength of the metal and have a crucial impact on the material’s performance. An excessively fast cooling rate may induce high thermal stresses within the forged part or result in internal stresses due to phase transformations. If these stresses surpass the strength of the forged piece, it could lead to cooling cracks, compromising the integrity and lifespan of the material [61]. Therefore, the air cooling rate is moderate, making it suitable for post-forging cooling of most titanium alloys [4]. Zhang et al. [62] studied how various cooling methods influence the room temperature tensile strength of the Ti-6.5Al-4.0Mo-4.0Zr-2.0Sn-1.0W-0.2Si alloy following isothermal forging. This research indicated that water quenching provided the highest tensile properties, with oil quenching ranking second and air cooling the lowest; however, compared with air cooling, the plasticity of the forging after oil quenching and water quenching is significantly reduced, which is associated with the degree of dispersion of the secondary α phase precipitates within the original β grains and the density of interfaces.

**Figure 8:**
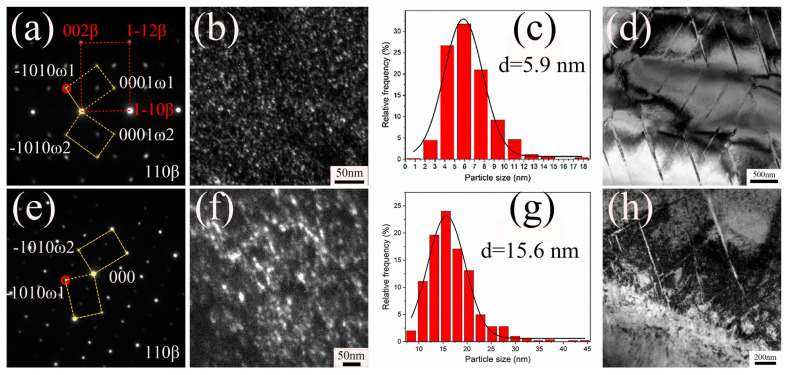
TEM image of Ti−5Mo−3Cr−Fe−3Zr alloy after cooling: (**a**–**d**) water quenching and (**e**–**h**) air cooling. (**a**,**e**) Record the diffraction pattern of the selected region along the [110]_β_ region axis. (**b**,**f**) Darkfield image using −1010ω1 points circled in (**a**,**e**). (**c**,**g**) Particle size distribution of the ω phase in water-quenched and air-cooled alloys. (**d**,**h**) Bright field images of the α″ [64].

In titanium alloys, varying cooling rates may result in the precipitation or transformation of different phases, consequently influencing the uniformity of the material’s structure. Zhu et al. [63] completed the mathematical modeling of the diffusion model of TA15 titanium alloy and studied the effect of the cooling rate of TA15 titanium alloy on the microstructure during heat treatment in the α/β phase zone. The titanium alloy was heated to 960 °C and kept warm for 30 min, then cooled at a rate of 9.5 °C/min, 60 °C/min, or 165 °C/min, respectively. Subsequently, the samples were water-quenched at 930 °C, 870 °C, 810 °C, or 750 °C to obtain microstructures for characterization. The findings indicated that the growth rate of the α phase is positively correlated with the cooling rate. With decreasing cooling rates, the grain distribution becomes increasingly uniform. Wang et al. [64] explored the effects of different cooling methods on the comprehensive properties of the ω phase and alloy after solution heat treatment in the β phase region of the metastable β titanium alloy (Ti-5Mo-3Cr-Fe-3Zr). Both water quenching and air cooling resulted in four variants, but only ω_1_ and ω_2_ were discernible. After air quenching and water cooling, the ω particles were uniformly dispersed in the β matrix (Figure 8b,f), and the average particle size of the ω phase after water quenching was 5.9 nm (Figure 8c). For air-cooled, the size was 15.6 nm (Figure 8g). Comparing Figure 8a,e, it is known that the diffraction intensity of the ω phase after water quenching is stronger, which indicates that air cooling is more helpful in forming more ω phase. The bright field image of the water-quenched alloy reveals that the α″ phase is needle-like. The morphology of the air-cooled α″ phase is similar to that of the water-quenched alloy (Figure 8h). Gao et al. [65] noted comparable microstructural features in the Ti-7Mo-3Cr-Fe alloy. Additionally, it is found that the growth of the ω phase in air cooling promotes the change in the deformation mechanism in the ω-vacancy channel. Therefore, in this study, an air-cooling method was proposed to control the growth of the ω phase, successfully combining high strength and high plasticity. Wei et al. [66] conducted air cooling and water cooling on isothermally forged TC25 (Ti-6.5Al-2Zr-2Sn-2Mo-1W-0.2Si) titanium alloy discs, revealing that air cooling yielded a coarse lamellar structure with large α phases, whereas water cooling resulted in a fine, intertwined basket-weave structure, resulting in variations of mechanical properties.

The cooling rate following forging greatly influences grain size and the formation of precipitate phases. The type of alloy, cooling method, applicable forging stage, and the cooling method after forging affect the final properties of the alloy. Thus, for titanium alloys with varying types and microstructural requirements, the cooling methods and rates must be thoughtfully evaluated.

### 2.5. Number of Isothermal Forgings

**Figure 9:**
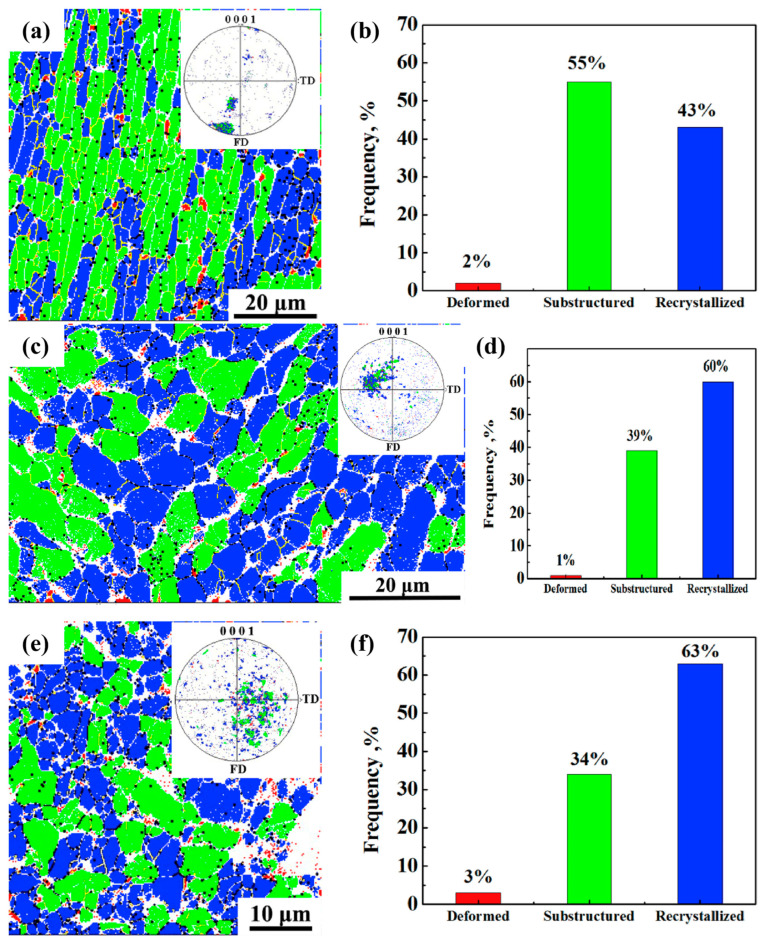
The degree of recrystallization of the Ti-6Al-4V alloy after isothermal forging the first step (995 °C, 1 × 10^−2^ s^−1^): (**a**) recrystallization image, (**b**) recrystallization fraction map; after the second isothermal forging step (950 °C, 1 × 10^−3^ s^−1^), (**c**) recrystallization image, (**d**) recrystallization fraction map; and after the third isothermal forging step (850 °C, 1 × 10^−3^ s^−1^), (**e**) recrystallization image, and (**f**) recrystallization fraction map [68].

The purpose of performing multiple isothermal forgings is to improve the microstructure and comprehensive properties of alloys. As the number of isothermal forging steps increases, the grains and phase structures within titanium alloys are likely to become progressively finer. Each forging step applies a specific amount of deformation to the material, resulting in grain breakage and the reorganization of phase structures. During multiple isothermal forging processes, this refinement effect may accumulate, thereby improving the mechanical properties of titanium alloys. Zhang et al. [67] studied the Ti-6Al-4V alloy and discovered that reducing the deformation temperature while increasing the forging cycle would lead to faster grain refinement and recrystallization in the billets. An annealed heterogeneous layered structure forms after the fourth forging step on the billets. Heterogeneous layered structures have superior strength-ductility synergies than equiaxed structures, and titanium alloys of this structure can achieve the high tensile strength of 1194 MPa and elongation of 13.1%. Zhang et al. [68] obtained a uniform equiaxed grain structure with an average grain size of 1.9 μm using a three-step forging method. Additionally, they explored the recrystallization and crystallization ratios of the Ti-6Al-4V alloy across various forging steps, as illustrated in Figure 9. As the number of isothermal forging steps increased, the recrystallization rate rose from 43% to 63%, while the crystallization ratio decreased from 55% to 34%. Additionally, the change in the direction of loading results in the spheroidization of the α phase during forging, with the proportion of high-angle grain boundaries rising from 48% to 71%. Tang et al. [69] compared the oxidation resistance of cast and wrought alloys. In contrast to cast alloys, the microstructure of the alloy with a two-step isothermal forging process changes from a coarse layered structure to a fine equiaxed crystal structure, causing the formation of smaller TiO_2_ particles on the alloy surface after oxidation, which effectively hinders further oxygen diffusion and results in improved oxidation resistance.

**Figure 10:**
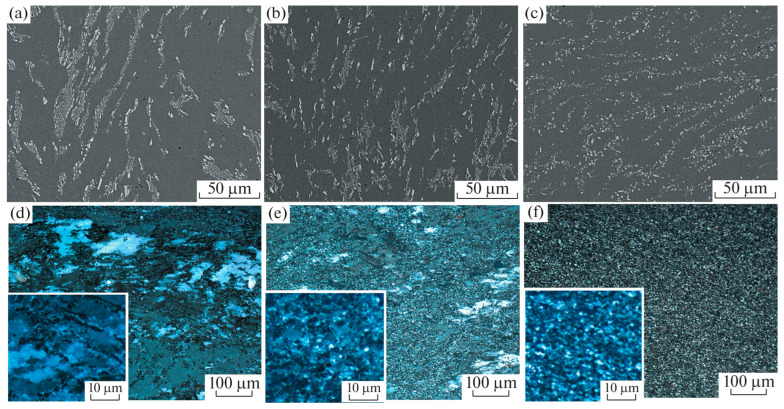
Microstructure of the Al-Mg-Fe-Ni-Zr-Sc alloy at 350 °C: (**a**,**d**) 2 cycles, (**b**,**e**) 4 cycles, and (**c**,**f**) 5 cycles [70].

Throughout the isothermal forging process, residual stresses within the material are progressively alleviated as deformation takes place. Multiple isothermal forging steps may help to further reduce the internal residual stress levels, enhancing the mechanical stability and service life of titanium alloys. The refinement of microstructure and decrease in residual stresses lead to favorable effects on the mechanical properties, specifically reflected in enhanced tensile strength, yield strength, and other mechanical metrics. Kishchika et al. [70] constructed stress–strain curves to study the multiple isothermal forging processes of the Al-Mg-Fe-Ni-Zr-Sc alloy. The research revealed that increasing the forging cycles from 2 (Figure 10a,d) to 4 (Figure 10b,e) and 5 (Figure 10c,f) resulted in a decrease in the average grain size from 1.3 ± 0.2 μm to 1.1 ± 0.1 μm. Through multiple forging steps, the yield strength of the titanium alloy increased by 60%, while the tensile strength improved by 20%. However, it is noteworthy that as the number of forging steps increases beyond a certain point, the enhancement in these properties may begin to saturate or even decline. This also verifies that multiple isothermal forging has a significant impact on grain refinement and performance improvement and provides a reference for the application of grain refinement of titanium alloys.

Multiple isothermal forging processes not only refine the grain size and reduce internal stresses but also contribute to a reduction in the number of defects in titanium alloys, such as porosity and cracks. These defects can negatively impact the mechanical properties of titanium alloys. Repeated forging combined with heat treatment can effectively reduce or eliminate defects in titanium alloys, significantly improving their overall quality. Furthermore, the thermal cycles associated with the forging process contribute to the enhanced thermal stability of alloys. Therefore, increasing the number of isothermal forging cycles in a controlled manner can lead to optimal performance and quality outcomes.

## 3. Future Research Directions

(1) Mechanisms of deformation

The isothermal forging process lacks direct visualization, making it challenging to observe the deformation of the workpiece, which can only be inferred through displacement measurements. The material’s deformation is driven by pressure control, complicating accurate assessments of the true microstructural changes occurring during the process [71]. To address this challenge, finite element simulations are necessary to continuously enrich the database, allowing neural algorithms to predict microstructural evolution with greater accuracy during deformation. Understanding the mechanisms of isothermal forging at the microstructural level can facilitate better control over the microstructure, ultimately enhancing alloy performance and informing industrial applications.

(2) Theory of plastic flow

The isothermal forging process currently lacks a specific plastic flow theory for titanium alloys that can accurately predict their high-temperature deformation behavior. Developing such a theory would not only reduce production costs but also facilitate precise control over titanium alloy forging. Most existing research primarily focuses on the study of forged specimens, with relatively few investigations dedicated to the underlying plastic flow theory. Insights from other studies on the superplasticity of titanium alloys can also be leveraged to enhance the understanding of the superplasticity of isothermally forged titanium alloys [72,73]. Furthermore, controlling the strain rate to achieve optimal superplasticity during titanium alloy deformation remains a key focus in isothermal forging research.

(3) Development of intelligent technologies

Future research is anticipated to employ finite element simulation techniques along with complex physical models to generate training data for neural networks, which will predict potential outcomes [74]. These predictions can then provide feedback to the finite element models, effectively guiding the isothermal forging process for high-performance titanium alloys. This intelligent technology can be applied in structural design, process design, and the optimization of new high-performance titanium alloy materials. For example, it can forecast complex structural responses in structural mechanics and simulate fluid flow, heat transfer, and other phenomena in fluid mechanics to optimize fluid system designs. Therefore, if real-time characterization technologies for the micro-forming features of complex components in isothermal forging can be developed, it would enable effective online prediction and dynamic regulation of both macro- and micro-evolution. This would facilitate an integrated approach to forming and formability while advancing the automation of process applications.

(4) Development of nanocrystalline materials

Nanocrystalline materials exhibit excellent plasticity and anti-aging properties [75,76]. If raw materials can be maintained at the nanoscale and utilized in isothermal forging, this could reduce the temperature and pressure required for the process, leading to enhanced properties for titanium alloys and addressing the challenge of achieving the high temperatures necessary for forging. Furthermore, the integration of nanocrystalline materials in isothermal forging technology research could facilitate the development of various properties in titanium alloys. Their unique characteristics hold significant value in both theoretical research and practical applications.

(5) Development of composite materials

Currently, the service temperature of titanium alloys is typically limited to around 600 °C [77]. Beyond this temperature, their heat resistance significantly diminishes, and achieving a balance between thermal strength and stability becomes challenging. This leads to a marked decline in oxidation resistance and fatigue performance, as well as an increased risk of “titanium fire”, which has long posed a significant safety threat to engine integrity. Consequently, research into new materials such as TiAl alloys and SiCf/Ti composites has gained considerable attention from researchers [78]. Existing composite materials commonly utilize reinforcements such as La_2_O_3_, SiC, TiC, TiB_2_, and TiB, which can effectively reduce thermal residual stresses during composite fabrication, resulting in lightweight titanium-based composites with excellent creep resistance [79,80]. Therefore, to ensure that these forgings meet the performance and safety requirements of aerospace applications, the development of high-performance titanium alloy composites through isothermal forging processes is essential.

## 4. Conclusions

Isothermal forging near-net shaping offers distinct advantages for metals characterized by high deformation resistance and limited deformation temperature ranges. This paper analyzes the forming characteristics of titanium alloys, clarifies the feasibility of isothermal forging for near-net shaping, discusses the influence of process parameters on microstructure and properties, and outlines future research directions. The following conclusions can be drawn:

(1) Temperature is the primary factor in isothermal forging. It significantly affects the phase transformation of titanium alloys, leading to the development of various microstructural morphologies and grain sizes. To enhance the toughness and strength of titanium alloys while minimizing the formation of secondary α phases and preventing grain coarsening, it is advisable to select a higher forging temperature whenever feasible. For example, in the single-phase region, the forging temperature is controlled to be 50 °C above the phase transition point, while in the two-phase region, it is controlled to be 30~50 °C below the phase transition point.
(2) The strain rate impacts grain size, microstructural uniformity, and the phase transformation process, thereby affecting the plasticity, fracture toughness, and strength of titanium alloys. High strain rates can lead to microstructural inhomogeneity and stress concentration, as well as alter the grain texture. Therefore, a relatively low strain rate should be selected to ensure microstructural uniformity and grain refinement. A strain rate of 0.001~0.0001 s^−1^ is typically chosen.
(3) Deformation amount influences the quantity of primary α phase, the thickness of the secondary α phase, and the dynamics of the recrystallization process. Significant deformation enhances dynamic recrystallization, leading to a finer and more uniform microstructure in titanium alloys. This refinement helps to improve the comprehensive properties and machinability of titanium alloys. Thus, the design of deformation should focus on increasing the deformation amount per cycle as much as possible without causing cracks. Therefore, the deformation amount is controlled within approximately 60~80%.
(4) The cooling method and cooling rate following forging play a crucial role in determining the properties of titanium alloys. These factors not only influence the content of precipitate phases but also contribute significantly to grain refinement during the forging process. Therefore, they should be optimized based on the specific performance requirements of the titanium alloy. Air cooling is currently the most widely used cooling method and is suitable for most titanium alloys.

Moreover, multiple-pass isothermal forging effectively facilitates grain refinement and reduces internal stress while decreasing the number of defects within titanium alloys, thereby enhancing their overall quality. The thermal cycles experienced during the forging process also improve the thermal stability of the alloys. Consequently, it is essential to appropriately increase the number of isothermal forging cycles to achieve optimal material performance.

## Figures and Tables

**Table 1:** Classification of titanium alloy microstructures with corresponding manufacturing processes and performance advantages.

Microstructure Type and Optical Microstructure		Manufacturing Processes [4]	Performance Advantages [4]
Equiaxed microstructure [5]	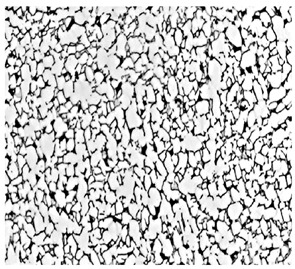	α + β forging Cross-β forging	High room-temperature strength Good plasticity High impact toughness Good fatigue limit Excellent high-temperature performance Good instantaneous plasticity
Duplex microstructure [6]	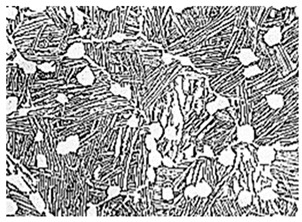	α + β forging Cross-β forging Near-β forging	High room-temperature strength Good plasticity Excellent high-temperature instantaneous plasticity
Basketweave microstructure [7]	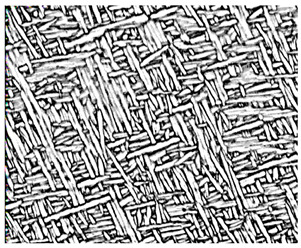	β forging Quasi-β forging	High room-temperature strength Good fracture toughness Excellent high-temperature performance Good instantaneous plasticity High creep resistance
Lamellar microstructure [8]	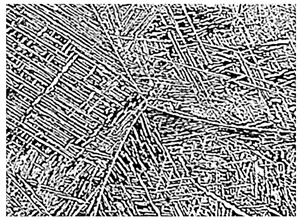	β forging	High room-temperature strength Good fracture toughness High long-term strength Excellent creep resistance

**Table 2:** Microstructural evolution mechanism of titanium alloy [53].

Alloy Type	Evolutionary Mechanisms
Near-α duplex alloys	Dynamic phase transitions
Ti-5Al-3V	The transition process from metastable β phase to steady α phase
Ti-6.5Al-1Mo-1V-2Zr	The content change in the primary α phase caused by deformation
Ti-5Al-2.5Sn	Dynamic phase transitions
Ti-6Cr-5Mo-5V-4Al	Dynamic recrystallization
Ti6.5Al-2.5Sn-9Zr-0.5Mo-1Nb-1W-0.25Si	Recrystallization promotes grain refinement
Ti-6Al-4V	Dynamic recrystallization of β phase spheroidization of layered α phase, and dynamic recrystallization of α phase
Ti-4Al-2.5V-1.5Fe	Subgrain boundaries transform small-angle grain boundaries into large-angle grain boundaries

## Data Availability

Written informed consent has been obtained from the patients to publish this paper.
